# Construction of an adherence rating scale for exercise therapy for patients with knee osteoarthritis

**DOI:** 10.1186/s12891-018-2200-x

**Published:** 2018-07-27

**Authors:** Jianji Wang, Long Yang, Qingjun Li, Zhanyu Wu, Yu Sun, Qiang Zou, Xuanze Li, Zhe Xu, Chuan Ye

**Affiliations:** 1 Department of Orthopedic Surgery, Affiliated Hospital of Guizhou Medical University, Guiyang, China; 2 Center for Bioprinting and Biomanufacturing, Guizhou Medical University, Guiyang, China; 3 Center for Tissue Engineering and Stem Cells, Guizhou Medical University, Guiyang, China; 4 China Orthopedic Regenerative Medicine Group (CORMed), Guiyang, China

**Keywords:** KOA, Exercise therapy, Adherence, Randomized controlled trial, Logistic regression analysis

## Abstract

**Background:**

Knee osteoarthritis (KOA) is one of the most common chronic diseases in the elderly and is the primary cause of the loss of motor function and disability in this population. Exercise therapy is a core, basic and matureand treatment method of treating patients with KOA. Exercise therapy is “strongly recommended” or “recommended” in the diagnosis and treatment guidelines of osteoarthritis in many countries, and most scholars advocate exercise therapy as the preferred rehabilitation method for KOA patients. However, poor long-term adherence is a serious problem affecting the therapeutic effect of this mature treatment. The objective of this study was to construct a concise and practical adherence rating scale (ARS) based on the exercise therapy adherence prediction model in patients with knee osteoarthritis.

**Methods:**

A binary logistic regression model was established, with the adherence of 218 cases of KOA patients as the dependent variable. The patients’ general information, exercise habits, knowledge, attitude, and exercise therapy were independent variables. The regression coefficients were assigned to various variables in the model, and the ARS was constructed accordingly. Receiver operating characteristic curves and curve fitting were used to analyse the effect of the ARS in predicting the adherence and to determine the goodness of fit for the adherence. The external validity of the ARS was examined in a randomized controlled trial.

**Results:**

The construction of the adherence model and the ARS included the following variables: age (1 point), education level (1 point), degree of social support (2 points), exercise habits (3 points), knowledge of KOA prevention and treatment (2 points), degree of care needed to treat the disease (1 point), familiarity with exercise therapy (4 points) and treatment confidence (3 points). The critical value of the total score of the ARS was 6.50, with a sensitivity of 87.20% and a specificity of 76.34%.

**Conclusions:**

A KOA exercise therapy adherence model and a simple and practical ARS were constructed. The ARS has good internal validity and external validity and can be used to evaluate the adherence to exercise therapy in patients with KOA.

## Background

In disease prevention or treatment, the World Health Organization (WHO) has defined adherence as the degrees of consistency of the behaviours of the patient concerning the diet, lifestyle and medication with the health care programme developed by the medical practitioner [1–3]. Meanwhile, the WHO has indicated that, compared with the development of a new treatment, the improvement in the patient’s adherence to the current mature treatment will result in greater health benefits [1]. Knee osteoarthritis (KOA) is one of the most common chronic diseases in the elderly and is the primary cause of the loss of motor function and disability in this population [4–6]. Exercise therapy is not only a mature [7] but also a core, basic and front-line treatment method of treating KOA [8–12]. Exercise therapy is “strongly recommended” [8] or “recommended” [9–12] in the diagnosis and treatment guidelines of osteoarthritis in many countries, and most scholars advocate exercise therapy as the preferred rehabilitation method for KOA patients [13, 14]. In recent years, large numbers of empirical studies have also provided evidence that exercise therapy can effectively relieve knee pain, reduce the rate of disability, improve knee function and improve the patient’s quality of life [15–18]. Therefore, improving adherence to this mature exercise therapy will enable KOA patients to reap more health benefits.

Poor long-term adherence is not only a serious problem affecting the health and quality of life of the population but also increases the economic burden of chronic diseases around the world [19, 20]. In practice, exercise therapy, similar to other chronic diseases, also has a serious poor adherence problem [21–24], which directly influences the therapeutic effect of this mature treatment. Therefore, an analysis of the risk factors of patient adherence and the interventions for these risk factors and for the improvement of adherence are important measures for ensuring the efficacy of this mature treatment.

In this study, considering the current poor patient adherence and low participation rate, an adherence prediction model was established with the adherence of the KOA patients as the dependent variable and the personal information, medical history and knowledge of KOA prevention and treatment as the independent variables. The values of the variables in the model were assigned based on regression coefficients to construct the adherence rating scale (ARS). This scale is expected to be able to provide early predictions of patient adherence and its risk factors, provide the basis for the development of personalized interventions and, ultimately, enable patients to achieve the greatest health benefits from exercise therapy.

## Methods

**Fig. 1:**
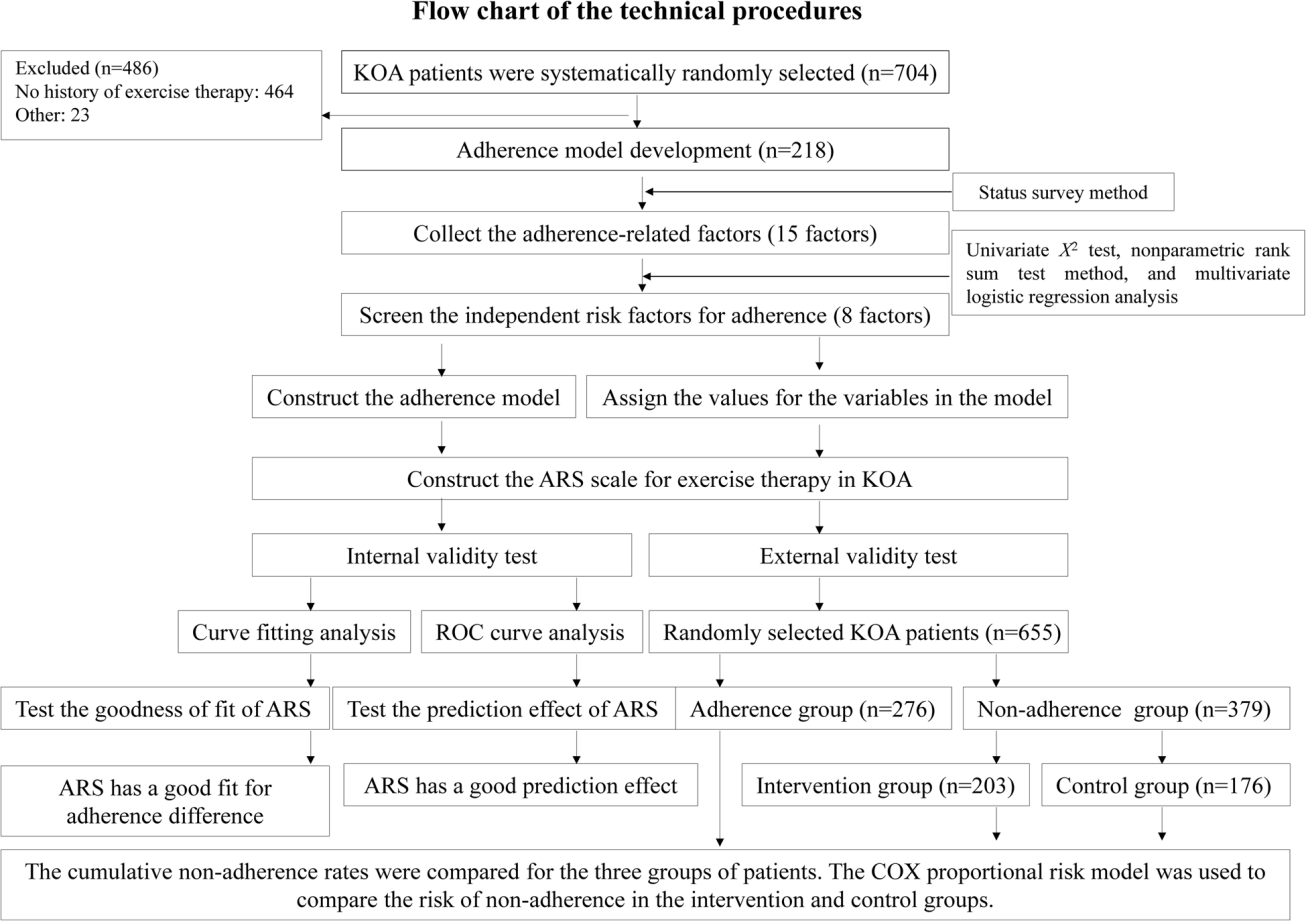
The technical design flow of the construction of the ARS for exercise therapy in KOA patients

In this study, an ARS for exercise therapy for KOA patients was constructed, and the internal validity and the external validity of this ARS were detected. The design flow is shown in Fig. 1.

### Construction of the ARS

Methods: The current status survey and multivariate logistic stepwise regression analysis were applied. Patients clinically diagnosed with KOA in the outpatient department of the affiliated hospital from October 2015 to July 2016 were collected as the study subjects. The sampling method was systematic random sampling, resulting in a total of 704 cases. Exercise therapy was not prescribed for 486 cases of the KOA patients and was prescribed for 218 patients. Among the 218 eligible KOA patients, there were 53 males and 165 females, with ages ranging from 38 to 80 years old. The current statuses of the 218 included patients were investigated and patients were classified into good adherence group and bad adherence group using Morisky scale to develop the adherence prediction model and the ARS.

The inclusion criteria for patients were as follows: (1) the patient diagnosis was in line with the Chinese Orthopedic Association (COA) osteoarthritis diagnosis and treatment guidelines (2007 edition) [11]; (2) the patient had performed or was performing exercises; (3) the patient provided informed consent; and (4) the patient showed clear consciousness, could correctly answer the questions and was willing to cooperate.

The exclusion criteria for patients were as follows: (1) the patient’s diagnosis was associated with severe medical diseases, such as severe heart, lung and brain diseases; (2) the patient had been diagnosed with any of the following mental disorders: cerebellar lesions, mental retardation, mental illness and cognitive disorders; (3) the patient experienced any of the following peripheral neuromuscular symptoms: limb muscle weakness, poor mobility or inability to perform autonomous activities; (4) the patient had showed no treatment history of exercise therapy (exercise was not recommended by the physician); or (5) the patient was unwilling to participate in the study.

In the development of the adherence prediction model, a survey with face-to-face interviews was conducted by 12 investigators using a structured questionnaire for the study subjects. The contents of the questionnaire included (1) personal information, such as age, gender, educational level and occupation; (2) daily exercise habits, such as whether the subject played sports and the daily exercise intensity; (3) the history of KOA and its treatment; (4) the knowledge of KOA prevention and treatment and the attitude and practices associated with the disease, that is KAP; (5) family, social factors, such as family members, and social support; and (6) exercise therapy factors, such as the complexity of the exercise therapy prescription and the familiarity of the exercise therapy. The questionnaire covered six areas and included more than 70 questions.

A binary logistic regression model was established with adherence as the dependent variable and personal information, exercise habits, exercise therapy familiarity, history of KOA and treatment history as the independent variables. While controlling some main influencing factors, the confounding factors were excluded. Other potential influencing factors were screened using univariate analysis. Factors showing *P* < 0.05 in univariate analysis and those with professional significance were included in the multivariate binary logistic regression analysis to finally determine independent influencing factors. The adherence prediction model was finally constructed.

To facilitate clinical application, this study assigned an appropriate value to each predictor based on its weight (which was calculated as the rounded number of the ratio of the regression coefficient of each factor and the minimum regression coefficient in the model) as the indicator to evaluate the adherence, thus constructing the ARS.

### Testing the internal validity of the ARS

The receiver operating characteristic (ROC) curve was used to analyse the total score of the patients’ adherence to exercise therapy, and the area under the curve (AUC) was calculated to evaluate the goodness of fit of the ARS, thereby determining the predictive effect of the total score on the adherence. Curve fitting was used to analyse the adherence score and the predicted possibility of the non-adherence rate correlation to assess the correlation and fitness of the ARS in distinguishing the adherence of exercise therapy.

### Testing the external validity of the ARS

For the method, a randomized controlled trial (RCT) was used. Patients who were newly diagnosed with KOA in the outpatient department from August 2016 to October 2017 were randomly selected, resulting in a total of 655 cases, including 156 males and 499 females. The inclusion and exclusion criteria were the same as those described above.

First, the ARS scores were obtained for all KOA patients (grouped by the pre-determined threshold), and the patients were divided into adherence and non-adherence groups based on their scores. Second, the cases in the non-adherence group were randomly divided into intervention and control subgroups by a random number table. The cases in the adherence, control and intervention groups were followed up for nine months to observe the changes in the non-adherence and the cumulative non-adherence of each group in each month. The interventions were given to the intervention group at the beginning of the third month of the treatment. The intervention measures included the knowledge of KOA exercise therapy developed based on a measurement domain in the ARS, the specific exercise measures and psychological counselling. Regular propaganda and education were provided for the control group. Propaganda and education measures included a complete exercise prescription, verbally informing patients that KOA needs exercise therapy and adhering to exercise therapy can alleviate their disease as well as improve their quality of life.

### Contents and methods of the exercise therapy

The contents of the exercise therapy and the methods of exercise used the guidelines of KOA treatment in China and other countries as references [12], which included lower limb muscle training, knee activity training and aerobic exercise. For the method used, according to the patient’s physical condition and the specific circumstances, each patient was given a corresponding exercise prescription. The goodness of adherence in this study was related to the patient’s individual exercise prescription.

### Exercise therapy and the adherence evaluation criteria

The method of adherence evaluation employed in this study was the Morisky scale [25]. Based on the Morisky scale, an adherence scale for KOA exercise therapy was developed, including four questions: (1) Have you ever forgotten to exercise once or more during exercise therapy? (2) Do you often not take exercise seriously? (3) If the joint pain and swelling symptoms are improved, do you stop exercising or reduce the number of exercises? (4) If you feel physically uncomfortable after exercise, do you stop exercising or reduce the number of exercises? For each question, the answer of “yes” was recorded as 1 point, and the answer of “no” was recorded as 0 points. The total score of the scale was 0–4 points. A total score of 0 points indicated that the adherence was good, and a total score greater than or equal to 1 point indicated that the adherence was poor. A higher score indicated worse adherence.

### Statistical methods

SPSS 19.0 statistical software was used for the data processing. The measurement data are presented as the means ± standard deviations, while the counting data are presented as frequencies. Dichotomous counting data such as gender, nature of occupation and exercise habit were tested using χ^2^ test; single-group ordered hierarchy data such as age and education level were tested using the nonparametric rank-sum test. Logistic stepwise regression analysis was used to analyse the factors influencing adherence. Differences with *P* < 0.05 were considered statistically significant.

## Results

### Construction of the ARS

In total, 704 cases of KOA patients were randomly selected, among which 486 cases (69.3%) were not recommended by the physician to perform exercise or did not meet the inclusion criteria, while 218 patients (30.7%) met the inclusion criteria. These 218 patients included 165 females (75.69%) and 53 males (24.31%), with an average age of 65.52 ± 15.14 years old. The shortest and longest courses of treatment for the patients were 14 and 229 days, respectively, with an average of 131.4 days.

For the univariate *x*^*2*^ analysis of adherence, the *x*^*2*^ test or the nonparametric rank-sum test was used to investigate the factors influencing adherence. Age, educational level, nature of the patient’s occupation, social support, lifestyle, exercise habits, the knowledge of KOA prevention and treatment, the degree of care needed to treat the disease, the familiarity of the exercise therapy and the complexity of the treatment regimen may have statistically significant impacts on the adherence (*P* < 0.05). The impacts of other factors on the adherence, such as gender, the length of disease history and the number of joints, were not statistically significant, as shown in Table 1.

**Table 1:** Univariate analysis of the influencing factors of adherence to KOA exercise therapy

Factor	Adherence		x^2^/Z	*P*
	Good	Bad		
Gender				
Male	22	31	0.038	0.846
Female	71	94		
Age #				
< 60	60	41	−4.979	< 0.001
60–75	24	44		
> 75	9	40		
Medical history #				
< 6	29	37	−0.332	0.740
6–12	31	41		
> 12	33	47		
Number of joints #				
Unilateral	29	30	−1.263	0.207
Bilateral	31	42		
Knee + other	33	53		
Education level #				
College and above	60	25	−6.734	< 0.001
High school	19	38		
Elementary school	14	62		
Nature of occupation				
Mainly mental work	69	72	6.427	0.011
Mainly physical work	24	53		
Social support				
Good	85	77	24.804	< 0.001
Fair	8	48		
Lifestyle				
Regular	79	57	35.181	< 0.001
Irregular	14	68		
Exercise habit				
Yes	60	47	15.459	< 0.001
No	33	78		
Knowledge of KOA prevention and treatment #				
Good	57	41	−4.830	< 0.001
Fair	29	45		
Poor	7	39		
Degree of care needed to treat the disease #				
Good	54	22	−7.492	< 0.001
Fair	33	41		
Poor	6	62		
Familiarity of the exercise therapy				
Familiar	88	82	26.162	< 0.001
Not familiar	5	43		
Treatment regimen				
Simple	74	70	13.212	< 0.001
Complex	19	55		
Medical staff-patient relationship				
Good	79	100	0.888	0.346
Fair	14	25		
Treatment confidence				
Good	80	100	1.343	0.246
Poor	13	25		

Note: # analysed using the nonparametric rank-sum test

For the multivariate logistic regression analysis of adherence, using the factors showing P < 0.05 in the univariate analysis including age, education level, nature of occupation, social support, lifestyle, exercise habit, knowledge of KOA prevention and treatment, degree of care needed to treat the disease, familiarity of the exercise therapy and complexity of the treatment regimen, as well as medical staff-patient relationship and treatment confidence with professional significance as independent variables, and the adherence as the dependent variable, multivariate logistic regression analysis was performed. The method of gradual entering was used, with entry and exclusion criteria of 0.05 and 0.10, respectively. The results were as follows. (1) The assigned values of the multivariate regression variables and the assigned weight are shown in Tables 2 and 3, respectively. (2) Age, educational level, social support, exercise habits, the knowledge of KOA prevention and treatment, the degree of care needed to treat the disease, the familiarity of the exercise therapy and the complexity of the treatment regimen were the independent influencing factors of adherence to KOA exercise therapy, while the nature of the KOA patient’s occupation could not be considered to have an independent impact on the adherence, as shown in Table 3.

**Table 2:** Assigned values of the multivariate regression variables for adherence to KOA exercise therapy

Variable	Assigned value
Adherence	Good = 0, poor = 1
Age	< 60 = 0, 60–75 = 1. > 75 = 2
Education level	College or above = 0, high school = 1, elementary school = 2
Nature of occupation	Mainly mental work = 0, Mainly physical work = 1
Social support	Good = 0, fair = 1
Lifestyle	Regular = 0, irregular = 1
Exercise habits	Yes = 0, no = 1
Knowledge of KOA prevention and treatment	Good = 0, fair = 1, poor = 2
Degree of care needed to treat the disease	Good = 0, fair = 1, poor = 2
Familiarity of the exercise therapy	Familiar = 0, not familiar = 1
Treatment regimen	Simple = 0, complex = 1
Medical staff-patient relationship	Good = 0, fair = 1, poor = 2
Treatment confidence	Good = 0, fair = 1, poor = 2

**Table 3:** Coefficients of the multivariate logistic regression for adherence to KOA exercise therapy

Variable/Factor	B	SE	Wald	*P*	OR	95%CI	Assigned value
Age	0.563	0.285	3.905	0.048	1.757	1.005–3.072	1
Education level	0.693	0.320	4.700	0.030	2.000	1.069–3.744	1
Social support	1.036	0.509	4.146	0.042	2.819	1.040–7.643	1
Exercise habit	1.740	0.426	16.713	0.000	5.698	2.474–13.123	3
Knowledge of KOA prevention and treatment	0.906	0.281	10.384	0.001	2.473	1.426–4.290	2
Degree of care needed to treat the disease	0.714	0.348	4.205	0.040	2.043	1.032–4.042	2
Familiarity of the exercise therapy	1.978	0.635	9.716	0.002	7.229	2.084–25.077	4
Treatment regimen	1.430	0.461	9.605	0.002	4.179	1.692–10.325	3
Constant	−3.745	0.564	44.095	0.000	0.024		

### Test of the internal validity of the ARS

**Fig. 2:**
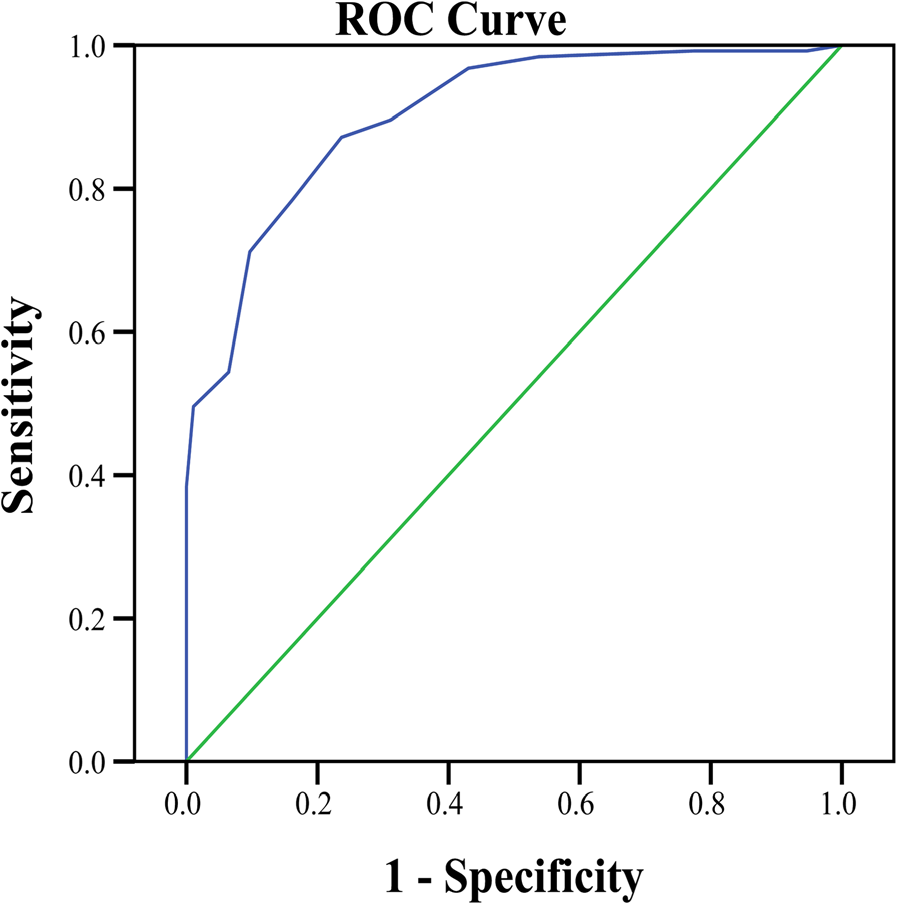
ROC curve of the adherence to KOA exercise therapy

An receiver operating characteristic (ROC) curve was used to analyse the predictive effect of the total score of the ARS on adherence. The AUC of the total adherence score was 0.903 (0.864–0.942), which was statistically significant, suggesting that the total score of the scale had a certain predictive effect on adherence. The critical value of the total score was 6.50, with a sensitivity of 87.20% and a specificity of 76.34%. The score of the adherence group was 4.43 ± 2.74, and the score of the non-adherence group was 10.91 ± 4.25, with a statistically significant difference (*t* = 12.854, *P* < 0.001) (Fig. 2).

**Fig. 3:**
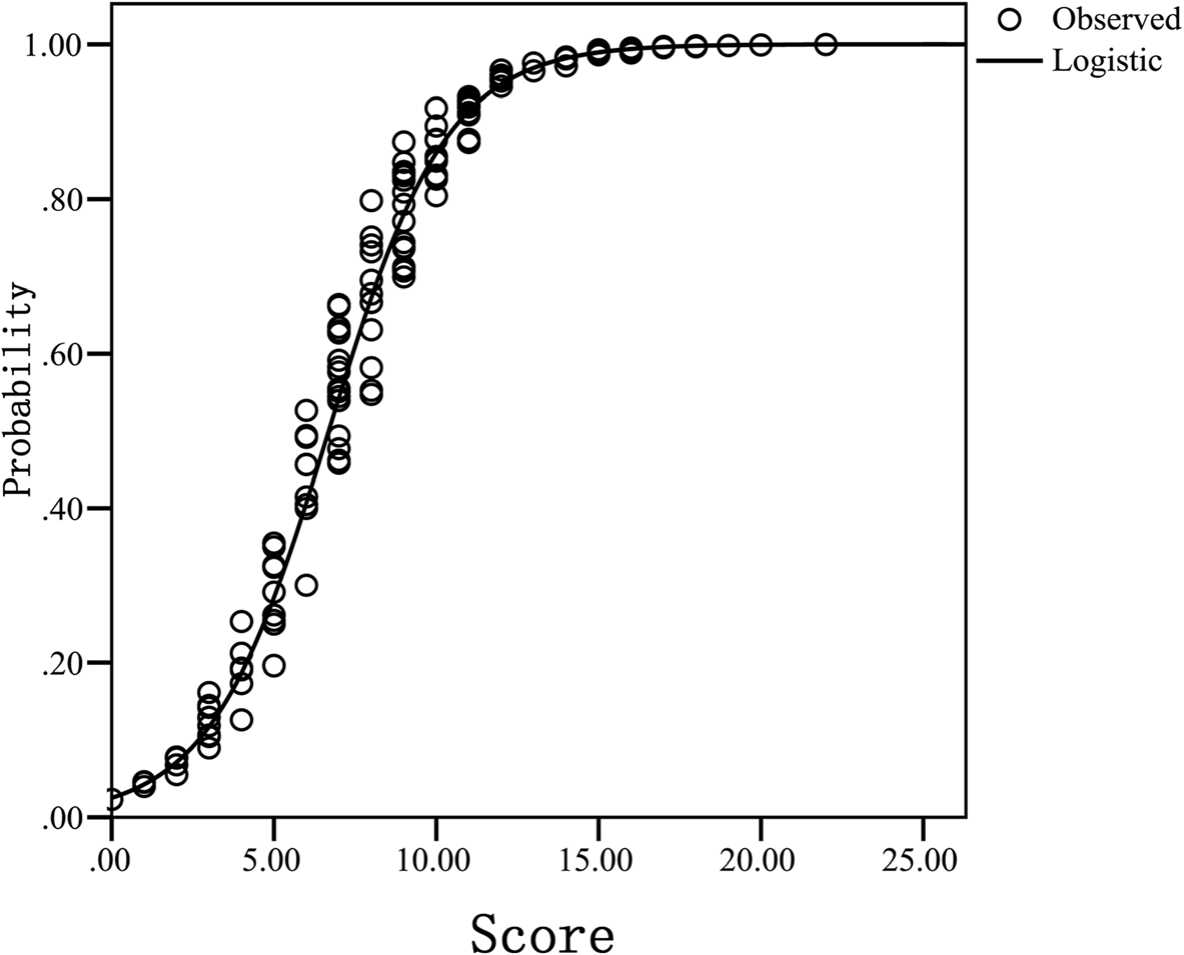
Curve fitting of the adherence score of KOA exercise therapy and non-adherence prediction probability

The Morisky scale (=0 for adherence; ≥1 for non-adherence) and the ARS (< 6.5 for adherence; ≥6.5 for non-adherence) (*x*^*2*^ = 89.786, *df* = 1, *P* < 0.001) were used to divide the study subjects into adherence and non-adherence groups. The differences between these two groups were compared, and the grouping results of the two methods were highly correlated (*x*^*2*^ = 89.786, *df* = 1, *P* < 0.001), suggesting that the ARS had a goodness of fit for the difference of exercise therapy adherence (Fig. 3).

### Test of the external validity of the ARS

There were a total of 655 KOA patients, including 156 males (23.82%) and 499 females (76.18%), with an average age of 64.28 ± 14.43 years. The subjects were divided into adherence (276 cases) and non-adherence (379 cases) groups according to their ARS scores (with a threshold of 6.5). Furthermore, the cases in the non-adherence group were randomly divided into intervention (203 cases) and control (176 cases) groups. There were no significant differences in the baselines for gender, mean age, mean follow-up time or treatment regimen among the three groups (*P* > 0.05).

**Fig. 4:**
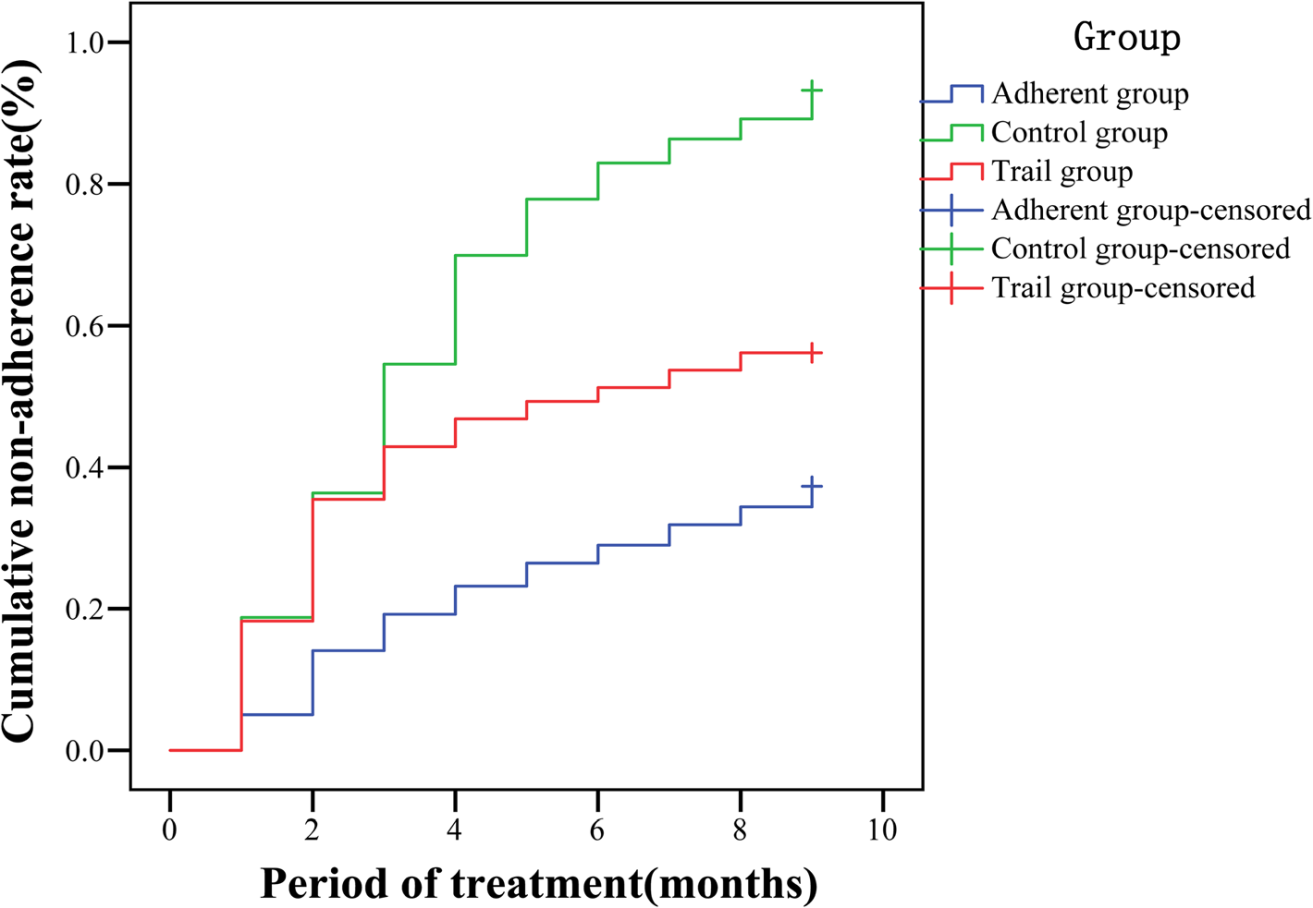
Cumulative non-adherence rates of the patients in the three groups at different treatment stages

The relationships of the cumulative non-adherence rates at different stages of the entire treatment course among the different groups are outlined in Fig. 4. There was no significant difference between the intervention and control groups in the first and second months of the treatment (*P* > 0.05), while in the third to ninth months of the treatment, the cumulative non-adherence rates of the intervention group were significantly lower than those of the control group (*P* < 0.05). This difference is because the individualized interventions for the intervention group were provided at the beginning of the third month of the treatment, indicating that the individualized interventions were effective and could significantly improve the treatment adherence of the KOA patients. The cumulative non-adherence rates of the control group at different stages of the entire treatment course were significantly higher than those of the adherence group (*P* < 0.05), indicating that the ARS had a good distinguishing effect on the actual adherence of the KOA patients; furthermore, the validity of the ARS was demonstrated. In addition, the cumulative non-adherence rates of the intervention group in the first two months of the treatment were higher than those of the adherence group, but the cumulative non-adherence rates in the last seven months of the treatment gradually approached those of the adherence group, though the differences were still statistically significant (*P* < 0.05), indicating that the intervention was effective, as shown in Table 4.

**Table 4:** Comparison of the non-adherence rates at different time points in each group

Group	1	2	3	4	5	6	7	8	9
Adherence group (*n* = 276)	14	35	44	52	59	67	74	79	82
Control group (*n* = 176)	33*	64*	94*	119*	133*	143*	149*	155*	159*
Intervention group (*n* = 203)	37*	72*	81*Δ	88*Δ	94*Δ	99*Δ	102*Δ	105*Δ	108*Δ
x^2^	25.662	38.637	64.190	97.270	114.301	125.300	128.161	130.837	138.317
*P*	< 0.001	< 0.001	< 0.001	< 0.001	< 0.001	< 0.001	< 0.001	< 0.001	< 0.001

Note: * Compared with the adherence group, *P* < 0.05; Δ compared with the control group, *P* < 0.05

The COX proportional hazards model was used to compare the onset risk of non-adherence for the intervention and control groups. The hazard ratio (HR) was 0.476 (95% confidence interval (CI): 0.373–0.607), and the difference of the intervention was statistically significant (*P* < 0.05), suggesting that the intervention was effective.

## Discussion

It is important to construct an ARS for KOA exercise therapy. Although the WHO has provided a theoretical framework for the influencing factors for adherence to chronic disease treatment, on the one hand, as time passes and with in-depth studies of chronic disease treatment models, the theory of adherence is also continuously developing and updating; on the other hand, the WHO adherence theory framework is constructed using generalized and universal influencing factors of chronic diseases, while specific descriptions for specific chronic diseases such as KOA exercise therapy are lacking. Therefore, this study constructed a special ARS for KOA as a specific disease.

In our study, the Morisky scale used for ARS construction was reliable in distinguishing good or bad adherence of KOA patients. Morisky adherence scale was first proposed by Morisky et al. in 1986 [26]. It is a universal measurement scale for medication adherence, and its items have little relevance with specific diseases or drugs. The report of WHO in 2003 mentioned this scale when discussing the measurement methods of adherence. In 2008, Morisky et al. added unintentional non-adherence (e.g. forgetting) and intentional non-adherence (e.g. deterioration of disease, worrying about drug adverse reactions) items on the basis of original scale, which increased the Cronbach’s α of scale to 0.83 [25]. Currently, according to disease characteristics of different specialties, researchers in different fields have constructed various adherence rating scales for the treatment of specific chronic diseases, such as scales for hypertension, pulmonary tuberculosis, acupuncture and moxibustion, and acquired immune deficiency syndrome (AIDS). Exercise therapy of KOA has general characteristics of chronic disease treatment. Therefore, we constructed the ARS for KOA exercise therapy on the basis of Morisky scale, and used this ARS to distinguish good or bad adherence of patients.

The ARS was constructed according to classical regression analysis, with good internal validity and coherence. Existing studies had mostly explored or elucidated the influencing factors for the adherence to exercise therapy. Few studies on the predictive model of risk factors for the adherence to exercise therapy and the research or application of quantitative evaluations of the variables have been reported. The eight independent variables in the adherence prediction model established in this study were obtained by screening more than 70 related factors; these variables covered several major measurement areas that affected adherence in the literature. Based on the adherence prediction model, the ARS was derived, and the ROC curve and curve fitting analyses were conducted for this scale. The scale had a good predictive effect and goodness of fit for the adherence. Therefore, the scale had good content validity, structural validity and distinguishing validity.

The ARS had good external validity and responsiveness. In general, an evaluation tool can be generalized and universal only after the evaluation of its external validity. To promote the ARS, this study investigated its external validity and responsiveness. In the test of external validity, interventions were given to the intervention group at the beginning of the third month to evaluate the effectiveness of intervention measures. Result showed that the cumulative non-adherence rates of intervention group after intervention were significantly lower than those of control group, indicating that the individualized interventions were effective and could significantly improve the treatment adherence of the KOA patients. Moreover, this also indicated that the ARS had good effect in judging the non-adherence risk factors of KOA patients, because the individualized intervention measures were developed based on the non-adherence risk factors of each patient evaluated by the ARS. In conclusion, the ARS is effective in distinguishing the actual adherence of KOA patients and identifying non-adherence risk factors.

This simple ARS is more suited to clinical practice. In analyses with univariate *x*^*2*^ testing and multivariate stepwise logistic regression, the adherence prediction model was ultimately determined. In general, the model can be directly used to predict the risk factors of patient adherence and to determine a given patient’s adherence, but to facilitate its use in clinical practice, this study simplified the model, that is, the ARS was constructed. The advantages of the ARS are as follows: (1) it provides direct and comprehensive quantifications of the risk factors of adherence to judge the patient’s adherence based on the total score; and (2) it allows for the quantification of each risk factor in the model to provide the basis for the development of personalized and targeted interventions.

The core of the intervention treatment is the intervention measures. Personalized and precise interventions can effectively reduce the rate of non-adherence. The eight items in the ARS can be broadly categorized into two classes: Class 1 includes short-term intervention items, such as the degree of care needed to treat the disease, the knowledge of KOA prevention and treatment, the familiarity of the exercise therapy and the confidence of the treatment; these items can be improved through appropriate interventions in a shorter term. Class 2 includes the non-short-term intervention items, such as the education level, social support and the habits of exercise; these items cannot be improved within a short time. Therefore, two principles are recommended in the use of this scale. First, key and non-key intervention principles: the intervention should be mainly conducted for items that can be intervened. Second, the principle of individuality: the specific risk factors and the number of items for each patient are different; therefore, the development of the intervention measures should follow the principle of individuality to achieve the medical precision goals.

This study has some limitations. No multi-centre research was performed. Second, the KOA cases were from China, and the education and medical knowledge levels of some cases were associated with local economic and education levels. Therefore, the development of ARS in other countries should be cautious when promoting.

## Conclusions

A KOA exercise therapy adherence model and an ARS were constructed by analyses of univariate factor *x*^*2*^ testing and a multivariate logistic stepwise regression. The ARS has good internal and external validity and can be used to evaluate the adherence to exercise therapy in patients with KOA.

## Abbreviations

ARS: Adherence rating scale
AUC: Area under the curve
CI: Confidence interval
HR: Hazard ratio
KOA: Knee osteoarthritis
RCT: Randomized controlled trial
ROC: Receiver operating characteristic
WHO: World Health Organization
